# Construction of Synthetic Probiotic Bacteria for In Situ Delivery of Anti-SARS-CoV-2 Nanobodies

**DOI:** 10.1007/s12602-025-10758-1

**Published:** 2025-09-11

**Authors:** Carolina E. Portero, Claire Smith, Yuxi Zhou, M. Raquel Marchán-Rivadeneira, Shiyong Wu, Yong Han

**Affiliations:** 1 Department of Chemistry and Biochemistry, Ohio University, Athens, OH 45701 USA; 2 Edison Biotechnology Institute, Ohio University, Athens, OH 45701 USA; 3 Center for Research on Health in Latin America (CISeAL)—Biological Science Department, Pontificia Universidad Católica del Ecuador (PUCE), Quito, 170143 Ecuador; 4 Honors Tutorial College, Ohio University, Athens, OH 45701 USA; 5 Department of Biological Sciences, Ohio University, Athens, OH 45701 USA; 6 Institute for Molecular Medicine and Aging, Heritage College of Osteopathic Medicine, Ohio University, Athens, OH 45701 USA

**Keywords:** Synthetic *Lactococcus lactis*, Surface display, RBD, SARS-CoV-2

## Abstract

SARS-CoV-2 viral infection can be inhibited by blocking the interaction between the viral spike protein and the human receptor angiotensin-converting enzyme 2 (hACE2). The development of specific spike inhibitors using nanobodies, the antigen-binding region of llamas’ antibodies, arose as a promising therapeutic method against SARS-CoV-2. However, one limitation of nanobodies is that they cannot be used directly in the human body due to their susceptibility to degradation. Bacteria-based delivery systems provide site-specific targeted action that can circumvent nanobody degradation. Here, we report the development of a genetically modified bacterium expressing anti-SARS-CoV-2 nanobodies that can inhibit the interaction between the hACE2 receptor and the receptor-binding domain (RBD) of the spike protein. *Lactococcus lactis*, a human symbiont probiotic bacterium, was selected to express nanobodies attached to their cell surface. Our data shows that FLAG-tagged anti-SARS-CoV-2 nanobodies were detected on the cell surface of recombinant *L. lactis *strains by flow cytometry and immunofluorescence without permeabilization. Furthermore, nanobodies are functional and can bind the RBD region from the spike protein in a dose-dependent manner. Inhibition of the hACE2-RBD interaction in cellular assays was quantified using a pseudotype lentivirus that mimics SARS-CoV-2 in an adaptation of the neutralization assay. Our results suggested that the recombinant bacteria can inhibit viral infectivity in more than 50% compared with a control without bacteria in a neutralization assay. These outcomes suggest that the engineered strain can be used in the future as a new therapeutic tool in COVID-19 prevention.

**Supplementary Information:**

The online version contains supplementary material available at 10.1007/s12602-025-10758-1.

## Introduction

The severe acute respiratory syndrome coronavirus 2 (SARS-CoV-2) is the infectious agent that causes the coronavirus disease 2019 (COVID-19), an airborne illness that led to a global pandemic in 2020 [1]. Although vaccines are available to prevent the spread of COVID-19, antiviral therapies remain essential for treating persistent and reemerging infections [2]. Establishing methods to block the entry of viral particles into human cells is crucial, as inhibiting the early stage of the SARS-CoV-2 life cycle can be an effective preventive measure [3].

Viral entry into human cells is mediated by the interaction of the spike (S) protein’s receptor-binding domain (RBD) and the human angiotensin-converting enzyme 2 (hACE2) receptor. The RBD within the S protein directly interacts with the hACE2 receptor on the surface of host cells. This interaction facilitates the attachment of the virus to the host cell, a necessary step for subsequent viral entry and replication. Therefore, the RBD of the spike protein has emerged as a key focal point for therapeutic purposes aimed at preventing or treating viral infections [4].

Recently, an alternative class of monoclonal antibodies called nanobodies has been developed to inhibit spike protein action with the hACE2 receptor [5]. Nanobodies (Nbs) are antibodies with a single variable domain located on camelid heavy-chain antibody (VHH) immunoglobulins responsible for antigen recognition [6]. Nbs offer a promising alternative to mAbs due to their small size, high affinity, strong stability, and ease of genetic engineering [7, 8]. Several techniques have led to the identification of almost 400 neutralizing nanobodies against SARS-CoV-2 [9, 10].

Specifically, Hou et al. (2020) described two nanobodies, H11-D4 and H11-H4, that block the interaction of RBD with ACE2 in vitro. Their structural information is available, and their binding mechanism has been described [11, 12]. Both nanobodies target an epitope adjacent to the ACE2-binding region on the RBD region with high affinity [12–14]. Furthermore, they were able to neutralize live wild-type virus, as well as the RBD region from Alpha, Kappa, and Delta variants, making them therapeutically promising [12, 15].

Although the future use of neutralizing antibodies to prevent and treat COVID-19 is a promising therapeutic application, nanobodies are prone to degradation because of their small size [16]. Thus, there is a need for a delivery system that can carry, protect, efficiently produce, and adequately deliver nanobodies to enhance their effectiveness*.*

In recent years, synthetic bacteria have emerged as a promising platform for producing and delivering therapeutic proteins [17, 18]. These engineered microbes offer key advantages over conventional drug delivery systems by enabling continuous, localized biosynthesis and controlled release of therapeutic molecules [19–22]. This approach has been widely explored in oncology and infectious disease treatment, where sustained in situ production of bioactive proteins can enhance therapeutic efficacy while minimizing systemic side effects [23–27]. Nanobody-based therapies face several challenges, including rapid clearance and the need for repeated administration [28]. Synthetic bacteria offer a potential solution by acting as self-sustaining bioengineered delivery vehicles. These microbes can colonize mucosal surfaces, such as the respiratory and gastrointestinal tracts, where they function as on-site bioreactors, continuously producing and displaying neutralizing nanobodies at the infection site [29, 30].

The probiotic bacterium *Lactococcus lactis* has been used to deliver biotherapeutics through the nasal and buccal mucosa [31, 32], the primary sites for the entry and infection of SARS-CoV-2 [33]. *L. lactis* is an acid-lactic [34], non-invasive [35], non-colonizing [36], gram-positive bacterium and a food additive with “generally regarded as safe” (GRAS) status by the US Food and Drug Administration (FDA) [37]. It can be genetically modified to display heterologous proteins [35]. Furthermore, recombinant proteins in *L*. *lactis* can be protected from degradation when they are anchored to the bacterial surface in a process known as protein surface display [38]. Through this process, the heterologous protein of interest is genetically fused to another protein that naturally binds to the cell surface and is exported outside the cells [39]. Given these characteristics, we hypothesized that *L. lactis* could be employed to produce neutralizing nanobodies against SARS-CoV-2, specifically targeting the interaction between the hACE2 receptor and the RBD region of the virus.

In this study, we developed a recombinant strain of *L. lactis* designed to deliver anti-SARS-CoV-2 nanobodies using a surface display system. This innovative approach involves using *L. lactis* as a delivery system for nanobodies to prevent and treat SARS-CoV-2 infections.

## Materials and Methods

### Bacterial Strain and Culture Conditions

The bacterial strain used in this study was *L. lactis* NZ3900 (MoBiTec GmbH, Germany) cultured without agitation in M-17 medium (Thermo Scientific™) supplemented with 0.5% lactose at 30 °C.

### Design and Construction of Recombinant Vectors

**Fig. 1:**
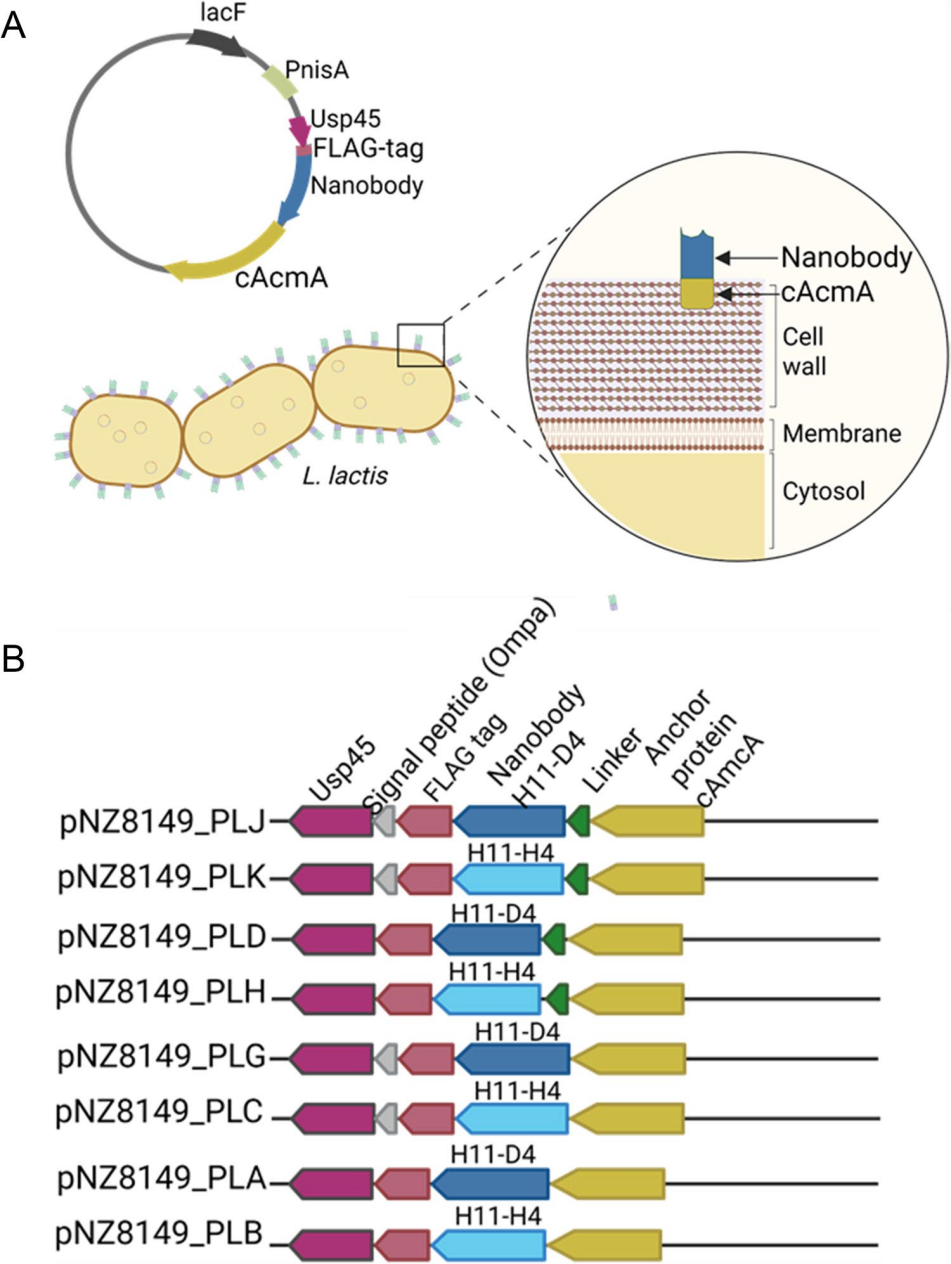
Design of *nanobody* surface display in *L. lactis*.** A** Genetically modified *L. lactis–*expressing nanobodies on its cell surface were developed by transformation with recombinant vectors. Illustration of the map of pNZ8149_PLA construct showing principal features: the selection marker LacF, the nisA promoter that controls protein expression, and the recombinant protein that includes the signal peptide Usp45, the FLAG-tagged nanobody, and the anchor protein cAcmA. Usp45 is cleaved in the exported mature protein, and the fusion protein is attached to the cell membrane by the cAcmA region*.* Created in BioRender. Portero, C. (2025) https://BioRender.com/c22d105 **B **Schematic representation of eight plasmid constructs with combinations of two different nanobodies (H11-D4 or H11-H4), a linker between the nanobody and the anchor, and an enhancer of the protein exportation (signal peptide OmpA). Created in BioRender. Portero, C. (2025) https://BioRender.com/a98b375

The design of surface display vectors was produced by the expression and export of a recombinant protein containing the selected nanobodies and an anchor domain that attaches to the cell surface (Fig. 1A) [38]. The sequence of the previously reported neutralizing nanobodies H11-D4 and H11-H4 that bind the receptor-binding domain (RBD) region of the spike protein was used in this study [12]. FLAG-tagged nanobodies were codon optimized for *L. lactis* and expressed using the food-grade plasmid pNZ8149 from the strictly nisin-controlled gene expression system [40]. The N-terminal Usp45 signal peptide was added to export the recombinant construct [41–43] via the Sec-dependent pathway, a metabolic route that translocates unfolded proteins across the cell membrane [44, 45]. Protein display was achieved by fusing the recombinant nanobodies to genes encoding non-covalent binding domains from a protein that naturally is attached to the *L. lactis* membrane, the C-terminus of N-acetylmuramidase (cAcmA) [46, 47]. A positive control using a reporter protein was used to validate the protein surface display design (Text S1, Fig. S1).

Eight vectors were constructed using the design described above accounting for a combination of three factors: (1) nanobody identity (H11-D4 or H11-H4), (2) presence/absence of a linker between the nanobody and cAcmA anchor domain, and (3) presence/absence of N-terminal signal peptide transporter enhancer (Fig. 1B). Additionally, two controls, a plasmid that produces the protein surface display of superfolder green fluorescent protein (sfGFP) (pNZ8149_Usp45_sfGFP) and a vector unable to export the nanobody, without the signal peptide Usp45 (pNZ8149_PLR), were constructed. All the plasmids utilized in this study are shown in Table 1.

**Table 1:** Bacterial strain and plasmids used in this study

Bacterial strain or plasmids	Relevant characteristics	Source or references
**Strain**		
*L. lactis* NZ3900	Used in food grade selection, contains the regulatory genes nisR and nisK integrated into the pepN gene and lactose operon without the lacF gene [48]	MoBiTec GmbH, Germany
**Plasmid**		
pNZ8149	Vector with a broad range, includes the lacF gene to restore the capacity to grow on lactose; nisA promoter to express the heterologous protein [48]	MoBiTec GmbH, Germany
pNZ8149_ Usp45___ sfGFP	Usp45 signal peptide, sfGFP, cAcmA anchor domain in pNZ8149. Superfolder GFP surface display plasmid	This study
pNZ8149_PLJ (PLJ)	Usp45 signal peptide, signal peptide OmpA, FLAG-tag, nanobody H11-D4, GGGGS linker, cAcmA anchor domain in pNZ8149. Nanobody surface display plasmid	This study
pNZ8149_PLK (PLK)	Usp45 signal peptide, signal peptide OmpA, FLAG-tag, nanobody H11-H4, GGGGS linker, cAcmA anchor domain in pNZ8149. Nanobody surface display plasmid	This study
pNZ8149_PLD (PLD)	Usp45 signal peptide, FLAG-tag, nanobody H11-D4, GGGGS linker, cAcmA anchor domain in pNZ8149. Nanobody surface display plasmid	This study
pNZ8149_PLH (PLH)	Usp45 signal peptide, FLAG-tag, nanobody H11-H4, GGGGS linker, cAcmA anchor domain in pNZ8149. Nanobody surface display plasmid	This study
pNZ8149_PLG (PLG)	Usp45 signal peptide, signal peptide OmpA, FLAG-tag, nanobody H11-D4, cAcmA anchor domain in pNZ8149. Nanobody surface display plasmid	This study
pNZ8149_PLC (PLC)	Usp45 signal peptide, signal peptide OmpA, FLAG-tag, nanobody H11-H4, cAcmA anchor domain in pNZ8149. Nanobody surface display plasmid	This study
pNZ8149_PLA (PLA)	Usp45 signal peptide, FLAG-tag, nanobody H11-D4, cAcmA anchor domain in pNZ8149. Nanobody surface display plasmid	This study
pNZ8149_PLB (PLB)	Usp45 signal peptide, FLAG-tag, nanobody H11-H4, cAcmA anchor domain in pNZ8149. Nanobody surface display plasmid	This study
pNZ8149_PLR (PLR)	FLAG-tag, nanobody H11-D4, cAcmA anchor domain in pNZ8149. Plasmid capable of producing nanobodies but unable to export outside the cytoplasm	This study

Briefly, surface-display plasmids were constructed via Gibson assembly (NEBuilder® HiFi DNA Assembly). DNA Fragments were synthesized as gBlock (Integrated DNA Technologies, USA) or obtained by PCR amplifications with Phusion High-Fidelity DNA Polymerase (NEB, M0530), and primers were designed with the NEBuilder assembly tool (https://nebuilder.neb.com/). Vectors were digested with SphI-HF, XbaI, SpeI-HF, and NcoI-HF restriction enzymes (New England Biolabs) and ligated with the fragments by the NEBuilder HiFi DNA assembly method (New England Biolabs), following the manufacturer’s protocol. The resulting plasmids were electroporated into competent *L. lactis* cells, and Lac + colonies were detected on Elliker agar [48]. The proper assembly of recombinant plasmids was confirmed by colony PCR and whole plasmid sequencing (Nanopore) by MWG Eurofins Genomics (Louisville, KY, USA). The primers and fragments used are shown in Table S1. The construction of each plasmid is described in Text S2. Sequences of the plasmids are available under the NCBI accessions (accession numbers pending).

### Recombinant Protein Induction

Overnight cultures of recombinant *L. lactis* were inoculated in 10 mL of M17 media with 0.5% lactose. Cultures were incubated at 30 °C without shaking until the optical density at 600 nm (OD_600_) reached 0.4. Then, the peptide nisin was utilized to induce protein production in the NICE system–based recombinant bacteria (NICE®) [40]. Nisin was added in a final concentration of 5 ng/μL in the genetically engineered *L. lactis*, unless stated otherwise. Cultures were incubated for 4 h at 30 °C without shaking.

### Western Blotting Analysis

Expression of FLAG-tagged nanobodies was visualized by western blot. Soluble proteins were extracted from induced recombinant strains according to the protocol described in the QIAexpressionist handbook with modifications [49]. Briefly, induced bacterial cultures were centrifuged at 3000 rpm for 10 min at 4 °C. After discarding the supernatant, the pellet was snap-frozen at − 80 °C and stored overnight. Bacterial cells were resuspended in 1 mL of lysis buffer (50 mM NaH2PO4, 300 mM NaCl, 5 mM imidazole, pH 8.0, 1X™ Mini EDTA-free Protease Inhibitor Cocktail (Complete-Mini, Roche)) and thawed in cold water. After one freeze (dry ice) and defrost cycle (cold water), the samples were incubated on ice with lysozyme (1 mg/mL) for 30 min. The lysate was sonicated twice for 5 s with 10 s pauses and centrifuged at 10,000 rpm for 30 min at 4 °C. Supernatants were collected, and total proteins were quantified using a DC protein assay kit, according to the manufacturer (Bio-Rad). Proteins were separated by 15% SDS-PAGE gel and transferred to a nitrocellulose membrane (pore 0.45 µm, Thermo Scientific, ID 88018). After incubation with Tris-buffered saline plus Tween 20 (TBST 1X), the membrane was blocked for 1 h in 5% w/v milk in TBST 1X. Primary antibody, rabbit anti-DYKDDDDK-tagged antibody (14793; Cell Signaling Technology), diluted in 1:1000, was incubated overnight at 4 °C. The secondary antibody, anti-rabbit immunoglobulin G (IgG) and horseradish peroxidase (HRP)–linked antibody (7074 s; Cell Signaling Technology), was incubated in 5% w/v milk in TBST 1X for 1 h. After three washes with TBST 1X, the membrane was developed in West Pico SuperSignal chemiluminescent substrate (Pierce). Images were acquired by Odyssey® Fc Imaging System (LI-COR Biosciences).

### Immunofluorescence Microscopy Without Permeabilization

To detect the proteins in the cell surface, immunofluorescence microscopy without impermeabilization was accomplished. In sterile conditions, 2 mL of the induced FLAG-tagged nanobody strain was centrifuged at 5000 rpm for 5 min at 4 °C and washed three times with phosphate-buffered saline (PBS 1X) for 5 min. The pellet was resuspended in 1.5 mL of PBS 1X, and 30 μL was placed on a cover slip to dry. A drop of paraformaldehyde (4% in PBS 1X) was added to the dried bacteria for 20 min, and then, it was washed three times with PBS 1X for 5 min. After 1 h of incubation with blocking solution (PBS 1X, FBS 50% v/v), anti-FLAG antibody (14,793; Cell Signaling Technology, 1:400 dissolved in antibody dilution buffer (PBS 1X/1% BSA/0.3% Triton™ X-100)) was incubated in a wet chamber overnight at 4 °C. Cells were washed three times with PBS 1X and stained for 1 h with the secondary antibody donkey anti-rabbit-IgG labeled with Alexa Fluor 488 (A-21206 from Thermo Scientific 1:800, dissolved in antibody dilution buffer). After three washes with PBS 1X, the coverslip was mounted with the ProLong™ Diamond Antifade Mountant (Thermo Scientific). Fluorescence microscopy was carried out using a Zeiss 510 laser-scanning microscope, LSM510 META (Thornwood, NY, USA) (GFP channel), and the digital images were processed using the open software ImageJ (RRID:SCR_003070).

### Flow Cytometry

Proteins in the cell surface were identified by flow cytometry based on Plavec et al. [47], with modifications. One milliliter of induced FLAG-tagged nanobody strains was centrifuged and washed with PBS 1X three times. The pellet was resuspended in 200 μL of diluted anti-FLAG antibody (14,793; Cell Signaling Technology, 1:400) and incubated for 2 h. After three washes with PBST (PBS 1X containing 0.05% Tween-20), the antibody donkey anti-rabbit-IgG labeled with Alexa Fluor 488 (A-21206 from Thermo Scientific, 1:800) was added and incubated for 2 h in the dark. Bacteria were washed three times with PBST and fixed in 1 mL PBS 1X, paraformaldehyde v/v 2% for 20 min. All antibodies were dissolved in PBS 1X, BSA w/v 1%, and incubated at room temperature with constant shaking. Samples were read with the flow cytometer BD Accuri™ C6 (Becton Dickinson, BD, Franklin Lakes, NJ, USA) using the FL1 channel with 20,000 gated events. Normalized median fluorescence intensity (MFI) of two independent assays was calculated.

### Determination of RBD Binding Capacity of Recombinant L. lactis by Indirect ELISA

Receptor-binding domain (RBD) binding capacity of the nanobody-surface *L. lactis* strains was determined by enzyme-linked immunosorbent assay (ELISA) [50]. Two milliliters of induced bacteria was washed three times with PBS 1X for 5 min. The bacterial cells were resuspended in PBS 1X, and the concentration was adjusted until it reached a value of 1.5 OD_600_ obtained in the microplate reader Cytation3 (BioTek, USA). Purified RBD in a final concentration of 0.75 ng/mL was incubated with 150 μL of the bacteria for 2 h at room temperature with gentle shaking. Samples were centrifuged for 7 min at 7000 rpm, and the supernatant was recovered without disturbing the pellet. Following the manufacturer’s recommendations, RBD concentration was obtained using an ELISA Kit (ELH-ACE2-1, Ray-Biotech). In this process, the pellet corresponds to the bacterial cells, and the RBD is attached to the cells, while the unbound RBD is in the supernatant. One-way ANOVA with Dunnett’s post hoc test was used to determine the differences.

In a second assay, different concentrations of the bacteria (pNZ8149-PLA strain) were used to determine binding properties. Induced bacteria were prepared as described above; after adjusting the OD_600_ to 1.5, five-fold dilutions were used. The samples were run in triplicate. Unpaired Student’s *t*-test between pNZ8149-PLA and the control for each concentration was calculated using GraphPad Prism 10.2.3.

### Spike Binding Detection by Immunofluorescence

To assess the RBD binding capacity of the pNZ8149-PLA strain, 2.03 × 10^9^ UFC of induced recombinant *L. lactis* were incubated with purified RBD in a final concentration of 0.75 ng/mL in 0.15 mL of PBS 1X for 2 h at room temperature. Cells were fixed, stained, and mounted as described in the immunofluorescence microscopy without permeabilization section. The primary and secondary antibodies utilized were SARS-CoV-2 Spike Protein (RBD) Polyclonal Antibody (PA5-114,451, Invitrogen, 1:800) and Alexa Fluor 488 labeled donkey anti-rabbit-IgG antibody (A-21206 from Thermo Scientific, 1:800), respectively. Images were captured by an AX10 observer Z1/7, Zeiss, fluorescence microscope.

### Human Cell Lines

The cell lines Human Embryonic Kidney Cells HEK-293 T (CRL-3216, ATCC) and HEK-293 T-hACE2 (NR-52511, BEI Resources) were cultured in antibiotic-free D10 growth medium, consisting of Dulbecco’s minimal essential medium (Corning™ 10013CV), 10% heat-inactivated fetal bovine serum (Gibco™, A5256701), and 2 mM L-glutamine (Gibco™, 25,030,081) at 37 °C with 5% CO_2_. When specified, D10 growth medium was supplemented with 100 U/mL penicillin and 100 mg/mL streptomycin (Corning®, 30–002-CI).

### Co-Culture of L. lactis and Human Cells

First, the co-culture conditions between *L. lactis* and human cells were determined. HEK-293 T cells were seeded on a poly-l-lysine-coated 96-well plate at a density of 1.25 × 10^4^ cells per well in 50 µL antibiotic-free D10 media (2.5 × 10^5^ cells/mL). An *L. lactis* culture was induced with nisin (final concentration 20 ng/mL) for 4 h as described in the “Recombinant Protein Induction” section; after three washes with PBS 1X, the bacteria were resuspended with PBS 1X to reach an OD_600_ of one, and eight successive ten-fold dilutions were made. In a 96-well plate, each bacterial dilution was mixed with 60 μL of antibiotic-free D10 media and incubated at 30 °C for 1 h before adding to the cells. Co-culturing was done with 15–20 h of the post-seeding HEK-293 T and 100 μL of the induced *L. lactis* in antibiotic-free D10 and incubated for up to 24 h at 37 °C with 5% CO_2_. Changes in pH were detected by measuring absorbance at 415 and 560 nm [51]. Incubation time and bacterial doses selected in the neutralization assay were chosen based on pH readout.

### Spike-Pseudotyped Lentivirus Production, Titration, and Viral Concentration

Lentivirus (LV) is a single-stranded RNA virus capable of infecting and integrating foreign DNA into human cells [52]. One of the LV envelope’s proteins can be replaced by the spike protein derived from SARS-CoV-2, changing the broad specificity of the LVs and allowing them to infect only cells expressing the hACE2 receptor [53, 54]; these viral particles are known as spike-pseudotyped LVs. The vesicular stomatitis virus (VSV)–pseudotyped virus packing system was used to prepare the spike-pseudotyped lentivirus using the protocol followed by Crawford et al. [55]. Briefly, HEK293T cells were co-transfected with plasmids HDM-Hgpm2 (NR-52517, BEI Resources), HDM-tat1b (NR-52518, BEI Resources), pRC-CMV-Rev1b (NR-52519, BEI Resources), and HDM_SARS2_Spike_del21_D614G (Addgene plasmid # 158,762) using BioT reagent (Bioland Scientific Catalog #B01-01). This system generates a pseudotype lentivirus containing one copy of the spike protein with tail truncation (D614G mutation). After 24 h of transfection, the medium was replaced with fresh, pre-warmed, antibiotic-free D10 media. Viral particles were harvested at 55 h post-transfection by collecting the supernatant, which was then filtered through a 0.45-μm SFCA low-protein-binding filter (Globe Scientific Inc., cat. no. 76516–626), aliquoted, and stored at − 80 °C until use. The positive and negative viral control particles were produced using the same procedures; however, the plasmid HDM_SARS2_Spike_del21_D614G was substituted by the VSV G plasmid (Addgene plasmid # 14,888) or transfection carrier DNA (Promega E4881), respectively. Titration was determined by measuring relative luciferase units (RLUs) in a serial dilution-based infection assay on HEK293T cells and HEK293T-hACE2 cells [55]. Briefly, 100 μL of viral dilutions was added to pre-seeded human cells in a poly-l-lysine-coated 96-well black plate (Corning, 3603); the cells were prepared in the same way as detailed in previous Sects. (2.5 × 10^5^ cells/mL). After 6 h of incubation, the medium was replaced with new D10 media with antibiotics. RLUs were measured when the cells had 100% confluency at 48–55 h post-infection using the Bright-Glo™ Luciferase Assay System (Promega). Ninety microliters of the cell media was discarded, and 30 μL of the Bright-Glo™ reagent was added. RLU was obtained after 2 min of incubation at RT, using the microplate reader Cytation3 (BioTek, USA) with no attenuation and integration time of 1 s. Viral concentrations were determined using lentivirus titration by qRT-PCR (Takara) according to the manufacturer’s instructions.

### Neutralization Assay Using Spike-Pseudotyped Lentivirus

To evaluate the inhibitory capacity of recombinant *L. lactis* strain with nanobody surface display on the hACE2-spike interaction at the cellular level, the viral infection of spike-pseudotyped LV on HEK293T-hACE2 cells was measured in the presence of *L. lactis* strains following the methodology described by Crawford et al. [55], with modifications. Bacteria and human cells were prepared as described in the “Human Cell Lines” section. Sixty microliters of each dilution of induced *L. lactis* re-suspended in PBS 1X was incubated with 60 μL of pseudotype LV (1.7 × 10^7^ RNA copies/mL) at 30 °C for 1 h in a 96-well plate. A 100 μL of the mixture was added to the HEK-293 T-hACE2 cells pre-seeded 15–20 h before at 2.5 × 10^5^ cells/mL. After 6 h of incubation at 37 °C and 5% CO_2_, the medium was replaced with 150 μL of D10 media with antibiotics. When the cells reached 100% confluence 48–55 h after infection, RLU were measured as previously described. To determine the background value, the average of the different negative controls was included: HEK-293 T-hACE cells, HEK-293 T-hACE and bacteria, HEK-293 T-hACE with negative carrier virus, HEK-293 T with pseudotype LV, pseudotype LV alone, and negative carrier virus alone. The positive controls, HEK-293 T-hACE cells with pseudotype LV, were included in duplicates in each column. The fraction infectivity for each experimental well was obtained with the following formula, using the positive control wells in the same column as the experimental well.

$$\frac{(RLUexperimental\;well\;-\;background)}{{\overline x\;(RLUpositive\;contol_1}\;-\;background,RLUpositive\;contol_2\;-\;background)}$$

As a first step, four biological replicates with a fixed concentration of *L. lactis* (4.5 × 10^6^ UFC/mL) were tested. ANOVA and post hoc test using Holm-Šídák’s post hoc test were calculated with GraphPad Prism 10.2.3. In a second assay, different concentrations of *L. lactis* strains were tested with a two-fold dilution starting with 4.5 × 10^6^ UFC/mL.

## Results

### Construction of Genetically Engineered L. lactis with Anti-SARS-CoV-2 Nanobody Surface Display

Recombinant strains of *L. lactis* with anti-SARS-CoV-2 nanobodies were developed using a surface display system by the expression and exportation of the selected nanobody, with an anchor domain that attaches to the bacterial cell surface. Eight vectors were constructed following the design shown in Fig. 1B.

**Fig. 2:**
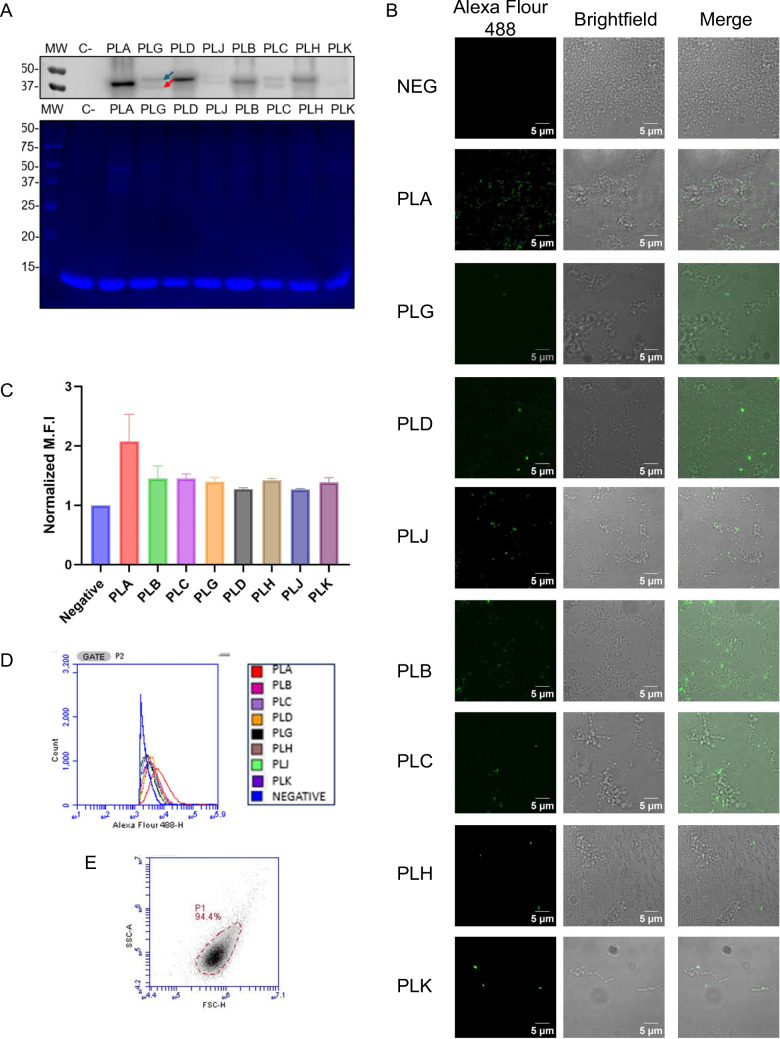
Nanobody surface display is present on recombinant *L. lactis*. **A** Visualization of FLAG-tagged nanobody by western blot (upper panel) and SDS-PAGE (lower panel). As a negative control (C −), *L. lactis* with backbone PNZ8149 was used. The blue arrow shows the protein with a signal peptide, while the red arrow shows the mature protein without a signal peptide. Total protein is shown by SDS-PAGE. **B** Nanobody surface expression by immunofluorescence microscopy without permeabilization. FLAG-tagged nanobodies are shown in green fluorescent (Alexa Fluor 488 Channel). Negative control has a nanobody unable to be transported outside the cell. **C–E** Flow cytometry of recombinant *L. lactis* displaying a FLAG-tagged nanobody. **C** Normalized median fluorescence intensity (MFI). **D** Scatter plot of the number of cells against fluorescence intensity. **E** Gate in side-scatter vs. forward-scatter graphic. The name of the strains is shown in its abbreviated form in all the figures, being derived from the plasmid PNZ8149

After induction, FLAG-tagged nanobodies were visualized with and without signal peptide by western blot in all the constructs (Fig. 2A). All nanobody constructs were genetically coded to produce the Usp45 signal peptide, and the presence of proteins without signal peptide is an indirect evidence of surface display, because the signal peptide is cleaved once the protein is translocated from the cytoplasm by the SEC machinery [56].

Surface display of the anti-SARS-CoV-2 nanobodies in *L. lactis* was confirmed by immunofluorescence microscopy without permeabilization and flow cytometry. As a negative control, a recombinant *L. lactis* strain unable to export the nanobody without a signal peptide was constructed. Microscopy showed the fluorescent staining of the FLAG-tagged nanobodies in all the constructs. In contrast, the negative control did not present fluorescence (Fig. 2B). Normalized median fluorescence intensity (MFI), obtained by scatter-gated flow cytometry (Fig. 2E), was higher in all recombinant strains than in negative controls, pNZ8149_PLA being the strain with a higher MFI (Fig. 2C). All the constructs presented more fluorescence intensity than the control (Fig. 2D). Our results demonstrated that our design produced recombinant *L. lactis* strains expressing anti-SARS-CoV-2 nanobodies on their cell surface.

### L. lactis Expressing Anti-SARS-CoV-2 Nanobodies Can Bind to the RBD Region of Spike Protein

**Fig. 3:**
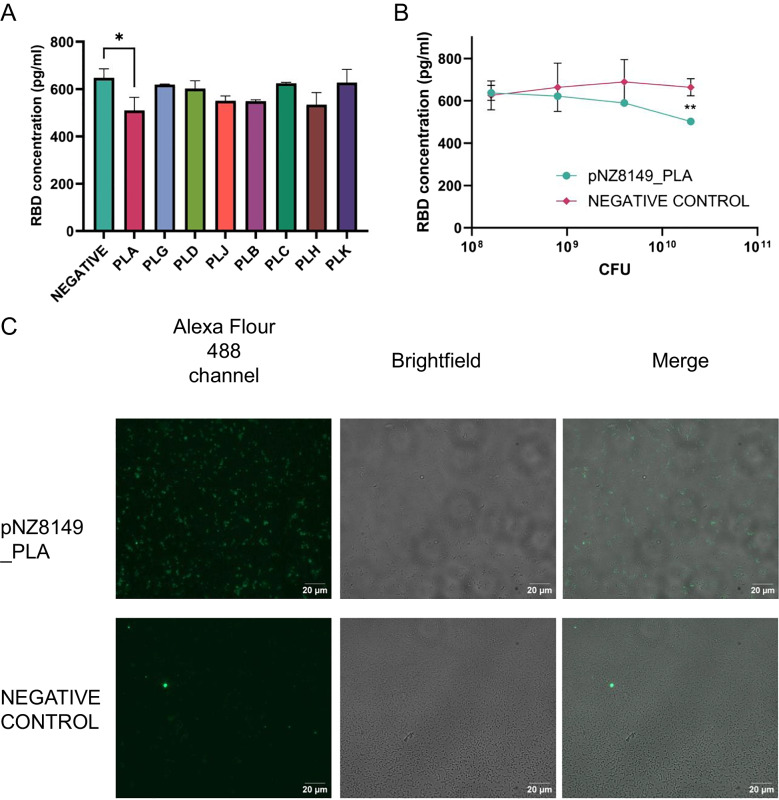
*L. lactis*–expressing nanobodies is able to bind the RBD domain of the spike protein on an indirect ELISA assay and immunofluorescence.** A** ELISA-based quantification of the RBD removal by recombinant bacteria. The name of the strains is shown in its abbreviated form. *P ≤ 0.05 (ANOVA-Dunnett’s test). **B** RBD removal increases with pNZ8149_PLA concentration, **P ≤ 0.01 (unpaired *t*-test). **C** The ability of *L. lactis*–expressing nanobodies to bind the RBD domain of the spike protein. Fluorescent signals were detected in the eGFP channel of the ZEN 2.6 pro software. Negative control is an *L. lactis* strain without a nanobody (PLR)

Receptor-binding domain (RBD) was incubated with bacteria to assess the binding capacity of the recombinant *L. lactis* variants. The mixture of bacterial cells and proteins attached to the cells was separated by centrifugation, and the unbound RDB was measured in the supernatant using ELISA. *L. lactis* with the plasmid pNZ8149 was used as a negative control. One strain presented statistically significant differences compared to the control, pNZ8149_PLA, which removed 21% more RBS than the negative control and was used in the following steps (Fig. 3A).

Incubation of increasing numbers of bacterial cells (pNZ8149_PLA) with RBD shows a direct relationship between RBD removal and the quantity of bacteria (CFU), being statistically significantly different from the control at 1.6 × 10^10^ CFU (Fig. 3B).

Immunofluorescence microscopy supported the binding of the RBD region to the *L. lactis* strain (pNZ8149_PLA) (Fig. 3C). After incubation of RBD with the bacterial cells, the RBD domain was identified in the H11-D4 surface-display *L. lactis* strain (pNZ8149_PLA), in contrast with the negative control. Overall, our data suggests that the nanobody in the cell wall of *L*. *lactis* is functional and can effectively bind the RBD region of the spike (S1) protein.

### L. lactis–Expressing Nanobodies Partially Inhibit RBD-hACE2 Interaction at the Cellular Level

**Fig. 4:**
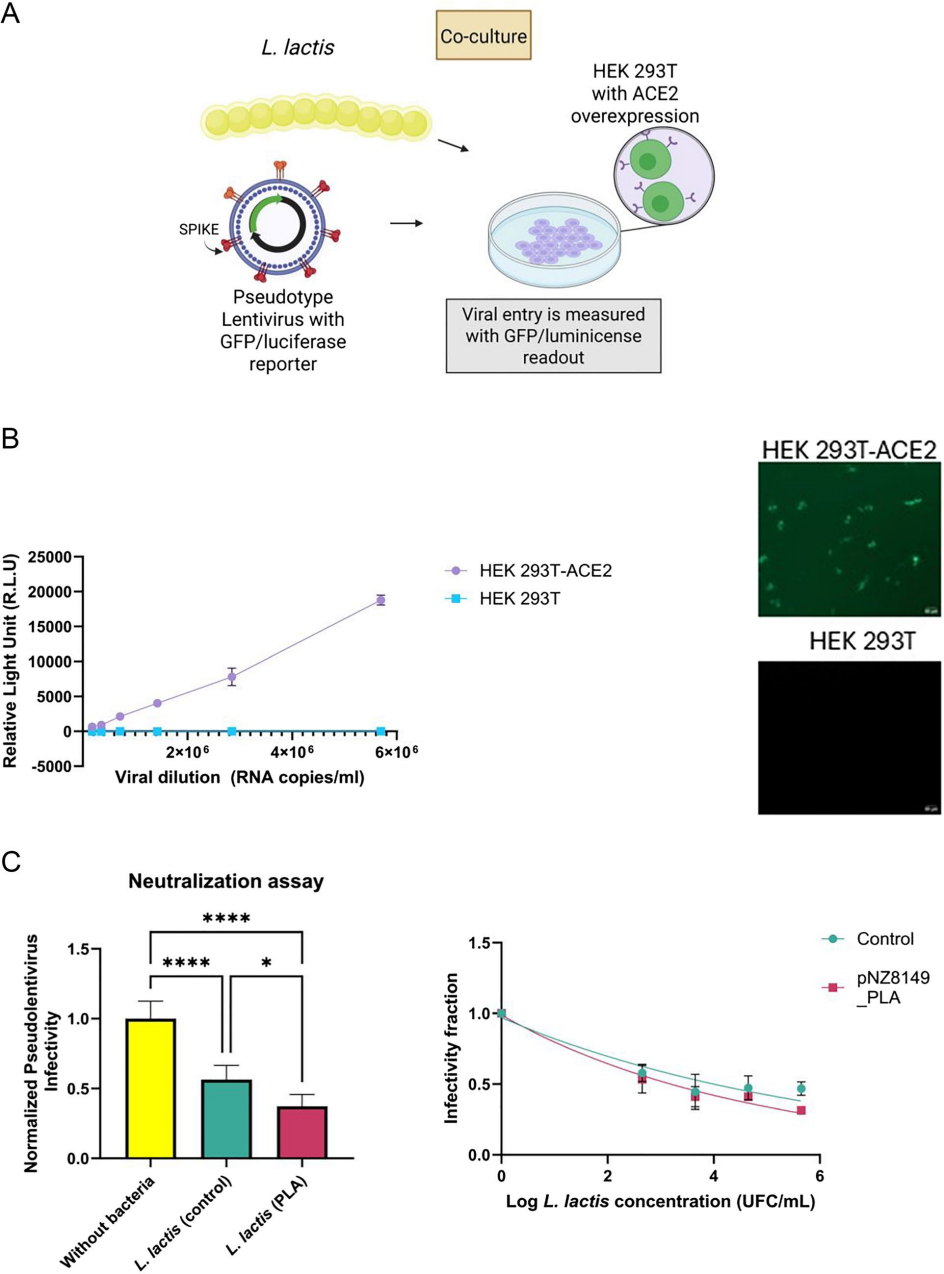
Recombinant *L. lactis* inhibits the RBD-hACE2 interaction. **A** Schematic representation of a neutralization assay. Viral infection induces the production of GFP and luciferase in the human cell expressing hACE2; inhibition by *L. lactis* decreases luminescence or fluorescence. Created in BioRender. Portero, C. (2025) https://BioRender.com/a50t807. **B** Titration of spike-pseudotyped lentivirus in cells with or without hACE2. At left: Detection of pseudo lentivirus titer by luminescence readout. Right: Human cells observed by fluorescence microscopy (GFP channel) 56 h post-infection of pseudotype lentivirus (1.7 × 10^7^ RNA copies/mL). The virus can only infect cells that express the hACE2 receptor. **C** In vitro pseudoviral infection assay. *L. lactis* produces a decrease in the viral infection. Left: Co-culture was done with HEK293T-hACE2 cells, spike-pseudotyped lentivirus, and two strains of *L. lactis* at a fixed concentration (4.5 × 10^6^ UFC/mL). *P ≤ 0.05, ****P ≤ 0.0001 (ANOVA-Holm-Šídák’s). Right: Neutralization assay with different concentrations of *L. lactis*

The inhibition of the interaction between RBD and hACE2 was assessed by a neutralization assay using spike-pseudotyped lentivirus. In this test, we evaluated whether the recombinant *L. lactis* strain (pNZ8149_PLA) can prevent viral infection in human cells. A pseudotype HIV-based lentivirus that mimics SARS-CoV-2 was created, which has the spike protein in its envelope and can only infect cells with hACE2 receptors. Lentivirus infection can be detected by inserting a reporter protein in the host genome (Fig. 4A). The neutralization assay was adapted to evaluate the response of the recombinant *L. lactis *strains with anti-SARS-CoV-2 nanobodies (Fig. S2). The selectivity of the infection of the spike-pseudotyped lentivirus constructed was evaluated, and it was shown to infect exclusively cells with the hACE2 receptor (Fig. 4B). Viral titration was achieved using luciferase as a reporter (RLU (relative light units)), and viral concentration (viral genome copy number/mL) was measured using RTq-PCR (Fig. 4B; Fig. S3). The concentration of 5.7 × 10^6^ (RNA copies/mL) was used in the following assays. Neutralization assay with *L. lactis* without nanobodies and the strain pNZ8149_PLA showed significant differences in comparison with the positive controls (cells with viral particles). *L. lactis* produced a decrease in the viral infection that was slightly enhanced when the nanobody was on the surface (Fig. 4C right). The neutralization assay showed that *L. lactis* strain pNZ8149**_**PLA can inhibit the viral entry into the cell in a dose-dependent manner (Fig. 4C left).

## Discussion

Bacteria-based delivery systems have arisen as a novel treatment approach against human diseases [57, 58]. Notably, the use of probiotic bacteria to prevent viral pathogenesis by capturing viral particles and toxins has been proposed [59–61]. In this study, the receptor-binding domain (RBD) protein of the SARS-CoV-2 virus attaches to the recombinant probiotic bacterium *Lactococcus lactis* and loses its infection capacity to the human receptor hACE2.

Specifically, we developed a series of recombinant *L. lactis* with nanobodies displayed on the cell surface. Nanobody surface display was confirmed by immunofluorescence microscopy without permeabilization and flow cytometry (Fig. 2B–D). Both techniques detect only proteins on the surface because the antibodies cannot cross the cell membrane without permeabilization [62]. Hence, the negative control, a recombinant *L. lactis* strain with intracellular nanobody production, did not show a fluorescent signal. Additionally, the location of the nanobodies is supported by our western blot results showing the presence of exported nanobodies. The signal peptide from the recombinant protein is cut after translocation from the cytoplasm to the membrane [63], producing a protein with a shorter size (Fig. 2A).

The nanobodies used in this study have been selected based on their therapeutic potential and the extensive information on their action mechanism [12, 64, 65]. H11-D4 and H11-H4 are affinity-matured nanobodies derived from a naïve alpaca VHH library that target to same epitope in the RBD region from the spike protein (Cluster 2) [12, 66]. Modeling data suggests that they can bind to different conformations of the spike protein, which may allow them to neutralize the virus at multiple stages in its life cycle [12, 66]. Their binding affinity has been compared under the same experimental conditions, allowing us to determine factors to improve our system in the future. H11-D4 and H11-H4 efficiency and binding capacity have been increased when other proteins were fused [12, 64], providing basic information to design the fusion protein with the anchor protein needed for the surface display.

Overall, our findings agree with previous research showing that binding between nanobody and RBD region in bacteria with surface display is influenced by the quantity of the target protein on the surface and the availability of its binding site [67]. Recombinant *L*. *lactis* PLA (pNZ8149_PLA) showed the most effective binding to the RBD region of the spike protein (Fig. 3B). Construct PLB (pNZ8149_PLB) is its closer analog differing by each other in the nanobody identity. It was expected that PLB would have a better performance because its nanobody, H11-H4, showed higher affinity to the spike protein than the H11-D4 nanobody (pNZ8149_PLA) when purified (*K*_D_ of 12 and 39 nM, respectively) [12]. However, pNZ8149_PLB presented a lower nanobody surface display than pNZ8149_PLA (Fig. 2C), decreasing the overall binding affinity.

Strains with H11-D4 nanobody consistently have better binding performance than their counterpart with H11-H4, with one notable exception, pNZ8149 PLH; its improvement is not related to the quantity of protein in the cell surface (Fig. 2C). pNZ8149 PLH has a linker between the nanobody and the anchor protein; linker regions may allow the nanobody to attain a better orientation in the cell wall and enhance the accessibility to its active site [38].

In a cell-based assay, we evaluated the capacity of *L. lactis* with an anti-SARS-CoV-2 nanobody surface display system to inhibit the interaction between the RBD region and the hACE2 receptor. The neutralization assay has been used to evaluate functional antibody responses of human plasma and purified nanobodies against the virus [68–70]. This study adapted it to assess the inhibition of viral infection in the presence of the *L. lactis *strains with anti-SARS-CoV-2 nanobodies (Fig. S2). Due to the limitation of the acidification of the culture media by the lactic acid production, the co-culture between *L. lactis*, the pseudotype lentivirus, and the human cells was conducted for 6 h (Fig. S2B). Our results show that viral entry into human cells can be detected with the modification proposed (Fig. S2C). However, the IC_50_ calculation is not possible to obtain because there is a limitation in the concentration of *L*. *lactis* used in this essay, which was not achieved to prevent changes in pH. Previous studies with the purified nanobodies H4D4 and H4D11 fused to the Fc domain of human IgG1 presented neutralizing activity against the SARS-CoV-2 virus (ND_50_ = 4–6 nM and 18 nM, respectively) when the virus and the nanobodies are incubated over 5 days [26]. Even though it is not possible to compare directly the two results, our findings are encouraging given the time limitation.

The recombinant bacterial strain can inhibit the RBD-hACE2 interaction in a neutralization assay; however, its effect is slightly better than *L*. *lactis* without surface display (Fig. 4C, D). According to the binding assay, non-recombinant *L. lactis* did not attach to the spike protein (Fig. 3A). In this conCtext, it is possible that *L. lactis* interacts with the cell and prevents the binding of the spike protein and the hACE2 receptor. Furthermore, indirect evidence suggests that *L. lactis* can be a natural inhibitor of the RBD-hACE2 receptor [71] and is able to grow near the tissues with high expression of the hACE2 receptors, such as the cells of the nasal [72] and salivary gland duct epithelium [73]. Alternatively, it is possible that the nisin produced by the autoinducer system NICE in *L*. *lactis* acts as an inhibitor. Docking data showed a significant binding affinity of nisin toward hACE2 [74]. Then, there may be intrinsic properties in *L. lactis* that allow natural interaction with the hACE2 receptor, potentially blocking the binding of RBD to the cell (Portero et al. In prep.).

*L. lactis* was chosen as a platform for nanobody production in this study based on its safety and feasibility to deliver recombinant proteins. *L. lactis* has been used to be used in clinical settings, showing its safety properties [18, 75–77]. Furthermore, the safety of a *L. lactis* strain genetically modified to secrete the human trefoil factor 1 (hTFF1) to oral mucosal tissue has been probed in a Phase Ib clinical trial for upper respiratory infection (URI) [78]. *L. lactis* can pass through the gastrointestinal tract (GIT) without colonizing and survive up to 36 h in humans; this transient nature contributes to its safety profile [36]. Despite being non-colonizing bacteria, there is considerable evidence to support the ability of *L. lactis* to deliver therapeutic proteins, including nanobodies, to the mucosal surfaces [22, 78–84]. In animal models, nasal and oral administrations of recombinant *Lactococcus* have been used to transfer antivirals to the mucosa [82, 85, 86].

Due to the reported natural inhibition of *L*. *lactis* and the increasing inhibition of engineering *L. lactis* strain with nanobody surface display, future work should focus on assessing the use of probiotics to block viral entry and improve the binding capacity of the nanobody. The insertion of anchors of varying lengths is one alternative because short anchors may produce steric hindrance in the binding site [87]. Another factor to consider is the identity of the nanobody. Signal peptide secretion efficiency depends on the protein structure; as a result, surface display can be enhanced with a different nanobody [88]. Several nanobodies have been developed and demonstrated higher performance than the nanobodies used in this study, targeting different epitopes and viral variants [89]. For this reason, a panel of different nanobodies should be considered in future steps.

In the present study, we constructed a NICE-based protein display system in *L. lactis*. The nisin-inducible (NICE) system, widely utilized in *Lactococcus lactis*, has been used to deliver recombinant proteins in mice [22, 90–92]. This approach is attractive from a therapeutic standpoint because, although the stability of protein production utilizing the NICE system has not been tested in humans, its expression is controlled by the food-safe inducer nisin [93]. Nevertheless, determining the human nisin dosage, plasmid stability, and biocontainment is required to contemplate a future practical application.

## Conclusion

In summary, we developed a probiotic bacterium that displays nanobodies on its surface, allowing it to bind to the spike protein and inhibit the interaction between the receptor-binding domain (RBD) and angiotensin-converting enzyme 2 (hACE2). This discovery has important theoretical and clinical implications, as the engineered strain represents a promising live biotherapeutic approach for COVID-19 prevention, with potential applications in mucosal drug delivery.

## Supplementary Information

Below is the link to the electronic supplementary material.

ESM 1 (DOCX 384 KB)

## Data Availability

No datasets were generated or analysed during the current study.
