# Supplementary material for: Construction of Synthetic Probiotic Bacteria for In Situ Delivery of Anti-SARS-CoV-2 Nanobodies
Source: Probiotics Antimicrob Proteins. 2025 Sep 11;18(3):4206–21. doi: 10.1007/s12602-025-10758-1 (PMC13176226; doi:10.1007/s12602-025-10758-1)
Supplement: Supplementary file 1 — (DOCX 384 KB) [file 12602_2025_10758_MOESM1_ESM.docx]

**SUPPORTING INFORMATION**

**Construction of synthetic probiotic bacteria to in-situ delivery of anti-SARS-CoV-2-nanobodies**

Carolina E. Portero^1,2,3^, Claire Smith^2,4^, Yuxi Zhou^1,2^, M. Raquel Marchán-Rivadeneira^2,3,5^, Shiyong Wu^1,2^, Yong Han^1,2^*

^1^Department of Chemistry and Biochemistry. Ohio University, Athens, Ohio 45701, USA.

^2^Edison Biotechnology Institute. Ohio University, Athens, Ohio 45701, USA.

^3^Center for Research on Health in Latinamerica (CISeAL) - Biological Science Department, Pontificia Universidad Católica del Ecuador (PUCE), Quito 170143, Ecuador.

^4^Honors Tutorial College, Ohio University, Athens, Ohio 45701, USA.

^5^Department of Biological Sciences, Ohio University, Athens, Ohio 45701, USA.

**Supplementary Text 1.** Microtiter plate assay and cell fractioning for superfolder green fluorescent protein (sfGFP) screening for GFP surface display. To evaluate the reporter protein production, *L. lactis* cultures with sfGFP surface display vectors were induced as described above with two concentrations of Nisin (5 and 20ng/µL). Fluorescent microscopy was carried out after 2,4, and 6h of incubation at 30C. One hundred µL of induced *L. lactis* culture were added to a 96 well plate black with clear bottom (Corning®). Fluorescence measurements were collected with a Citation 3 plate reader (BioTek) (excitation:485nm, emission: 528nm). Normalization was accomplish according to Overkamp et al ^1^. Treatments were compared with the two-tail Student’s t test.

To detect sfGFP surface display, proteins from different parts of the bacterial cell were isolated according to Davarpanah et al. ^2^ with modifications. In brief, 7 mL of induced recombinant *L lactis* culture were washed with PBS 1X by centrifugation. The pellet was dissolved in 1 mL of TES buffer 1X (10 mM Tris-HCl pH 8, 1 mM EDTA, 25% Sucrose) with lysozyme (5 mg/mL) and incubated per one hour at 37⁰C. After centrifugation (4,300 rpm per 10 minutes at 4⁰C), the proteins of the supernatant were extracted with trichloroacetic acid (TCA) and corresponded to the cell wall fraction. The pellet was washed with TES buffer 1X, resuspended in 500 µL of water, and frozen/thawed five times. After centrifugation (1h 30 h, 16,000 rpm at 4⁰C), the cytoplasmatic proteins were obtained via TCA extraction of the supernatant. The new pellet was resuspended in PBS 1X/SDS1% to isolate the membrane proteins. The sfGFP were visualized by western blot using rabbit monoclonal anti-GFP (2956T Cell Signaling Technology) as primary antibody, and anti-rabbit IGG hrp-linked antibody (7074s Cell Signaling Technology) as secondary.

**
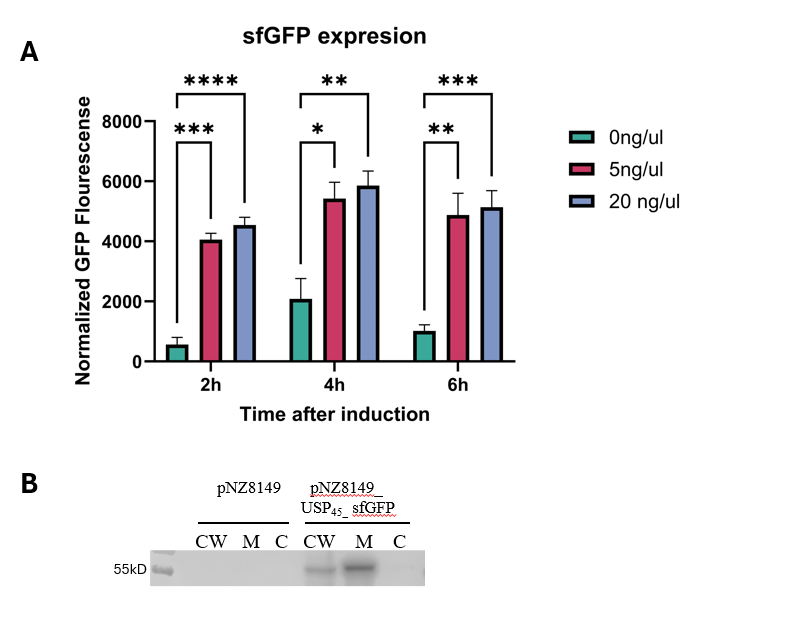
**

**Supplementary Fig. 1.** Reporter-based protein surface display system in *L. lactis****.*** Nisin was used to induce sfGFP-surface-display in recombinant *L. lactis* cultures. Non-induced *L. lactis* containing empty plasmid pNZ8149 was used as negative control. **A.** Time course of the normalized fluorescence of recombinant *L. lactis.* Two concentrations of Nisin were used. The data shown are averages of four technical replicates. Fluorescence plate readers were set at Excitation 485nm and Emission 528nm. **B.** sfGFP distribution in different cell compartments of recombinant *L. lactis*. Western blot using anti-GFP antibody was done on fractionated protein extracts from L. lactis. The assay corresponds to 4 h induction with 5 ng/µl of Nisin. CW: cell wall, M: membrane, C: cytoplasm.

**Supplementary Table 1**. Oligonucleotides and fragments sequencing

| **Oligonucleotide** | **Sequence** |
| --- | --- |
| Usp_45__fwd | aaataaattataaggaggcactcaccatgaaaaaaaagattatctcagc |
| Usp_45__flag_tag_rev | ttttttgcatgctttatcatcatcatctttataatcagcgtaaacacctg |
| H11-D4_fwd10 | gatgatgatgataaagcatgccaagttcaattagttgagag |
| H11-D4_rev10 | Tgctgccgcttttacttgaaactgttacttgag |
| nanobody_fwd2 | Tgatgatgataaagcatgcaaaaaaacagcc |
| nanobody_RV_cAcmA 2 | Ggtgctgctgccgctactagttttacttgaaactgttac |
| nanobody_fwd13 | gatgatgatgataaagcatgcaaaaaaacagccatcgc |
| nanobody_rev13 | Ctccaccccctttacttgaaactgttacttgag |
| cAcmA_fwd | Tgagctctacaaaagcggcagcagcaccacc |
| cAcmA_rev | agtggtaccgcatgcctgcagtaccttatttaatgcgcagatactggccaatcagaatgg |
| cAcmA_fwd2 | gtaacagtttcaagtaaaactagtagcggcagcagcaccacc |
| cAcmA_rev2 | gaaagcttgagctctctagaattatttaatgcgcagatactg |
| cAcmA_fwd10 | tcaaagaaagcttgagctctttatttaatgcgcagatactggccaatcagaatgg |
| cAcmA_rev10 | Ttcaagtaaaagcggcagcagcaccacc |
| cAcmA_linker_fwd14 | Ttcaagtaaagggggtggaggtagtggc |
| cAcmA _linker_rev14 | tcaaagaaagcttgagctcttctagaattatttaatgcgcagatactggcc |
| **Synthetized DNA fragments** |  |
| cAcmA | Agcggcagcagcaccaccaccaacaccaacaacaacagcggcaccaacagcagcagcaccacctataccgtgaaaagcggcgataccctgtggggcattagccagcgctatggcattagcgtggcgcagattcagagcgcgaacaacctgaaaagcaccattatttatattggccagaaactgctgctgaccggcagcgcgagcagcaccaacagcggcggcagcaacaacagcgcgagcaccaccccgaccaccagcgtgaccccggcgaaaccggcgagccagaccagcgtgaaagtgaaaagcggcgataccctgtgggcgctgagcgtgaaatataaaaccagcattgcgcagctgaaaagctggaaccatctgagcagcgataccatttatattggccagaacctgattgtgagccagagcgcggcgaccagcaacccgagcaccggcagcggcagcaccgcgaccaacaacagcaacagcaccagcagcaacagcaacgcgagcattcataaagtggtgaaaggcgataccctgtggggcctgagccagaaaagcggcagcccgattgcgagcattaaagcgtggaaccatctgagcagcgataccattctgattggccagtatctgcgcattaaa |
| USP45_sfGFP^1^ | aaataaattataaggaggcactcaccatgaaaaaaaagattatctcagctattttaatgtctacagtgatactttctgctgcagccccgttgtcaggtgtttacgct**agcaaaggagaagaacttttcactggagttgtcccaattcttgttgaattagatggtgatgttaatgggcacaaattttctgtcagtggagagggtgaaggtgatgctacatacggaaaactcacccttaaatttatttgcactactggaaaactacctgttccttggccaacacttgtcactactctgacctatggtgttcaatgcttttcccgttatccggatcacatgaaacggcatgactttttcaagagtgccatgcccgaaggttatgtacaggaacgcactatatctttcaaagatgacgggaactacaagacgcgtgctgaagtcaagtttgaaggtgatacccttgttaatcgtatcgagttaaagggtattgattttaaagaagatggaaacattctcggacacaaactagagtacaactataactcacacaatgtatacatcacggcagacaaacaaaagaatggaatcaaagctaacttcaaaattcgccacaacattgaagatggttccgttcaactagcagaccattatcaacaaaatactccaattggcgatggccctgtccttttaccagacaaccattacctgtcgacacaatctgccctttcgaaagatcccaacgaaaagcgtgaccacatggtccttcttgagtttgtaactgctgctgggattacacatggcatggatgagctctacaaa***agcggcagcagc*a |
| H11D4+linker^2^ | attataaagatgatgatgataaagcatgc**caagttcaattagttgagagtggtggaggcttaatgcaagcaggagggagtctccgcctatcttgtgctgtatctggcagaactttttcaaccgcagcaatgggttggtttcgtcaagctcctggtaaagaacgtgaatttgttgctgcaattcgttggtcaggtggttcagcttactatgctgattctgtcaagggacgattcacgatttctcgggataaagcaaaaaatacagtataccttcaaatgaactcattaaaatatgaagatacagcagtttattattgtgcaagaacagaaaatgttcgttcgttgttgagcgactatgccacttggccatatgattattggggacaaggaactcaagtaacagtttcaagtaaa**GGGGGTGGAGGTAGTGGCGGAGGTGGTAGTGGCGGGGGAGGATCTagcggcagcagcaccaccac |
| H11D4 block^3^ | ataaagatgatgatgataaagcatgcaaaaaaacagccatcgctattgccgtggcgcttgctggttttgctactgtcgcgcaggct**caagttcaattagttgagagtggtggaggcttaatgcaagcaggagggagtctccgcctatcttgtgctgtatctggcagaactttttcaaccgcagcaatgggttggtttcgtcaagctcctggtaaagaacgtgaatttgttgctgcaattcgttggtcaggtggttcagcttactatgctgattctgtcaagggacgattcacgatttctcgggataaagcaaaaaatacagtataccttcaaatgaactcattaaaatatgaagatacagcagtttattattgtgcaagaacagaaaatgttcgttcgttgttgagcgactatgccacttggccatatgattattggggacaaggaactcaagtaacagtttcaagtaaa**GGGGGTGGAGGTAGTGGCGGAGGTGGTAGTGGCGGGGGAGGATCTagcggcagcagcaccaccac |
| H11H4 block^4^ | Ataaagatgatgatgataaagcatgcaaaaaaacagccatcgctattgccgtggcgcttgctggttttgctactgtcgcgcaggct**caagttcaattagttgagagtggtggaggcttaatgcaagcaggagggagtctccgcctatcttgtgctgtatctggcagaactttttcaaccgcagcaatgggttggtttcgtcaagctcctggtaaagaacgtgaatttgttgctgcaattcgttggtcaggtggttcagcttactatgctgattctgtcaagggacgattcacgatttctcgggataaagcaaaaaatacagtataccttcaaatgaactcattaaaatatgaagatacagcagtttattattgtgcacagacgcattatgtcagctacttgttgagcgactatgccacttggccatatgattattggggacaaggaactcaagtaacagtttcaagtaaa**GGGGGTGGAGGTAGTGGCGGAGGTGGTAGTGGCGGGGGAGGATCTagcggcagcagcaccaccac |

^1^In underline: USP_45,_ in bold: sfGPF

^2^In bold: H11D4, uppercase: linker

^3^In underline: signal peptide OmpA, in bold: H11D4, uppercase: linker

^4^In underline: signal peptide OmpA, in bold: H11H4, uppercase: linker

**Supplementary Text 2.** Plasmids construction strategy

**pNZ8149_USP_45__ sfGFP**

**Fragment 1:** pNZ8149 was digested with NcoI HF (2550pb). **Fragment2:** Usp45_sfGFP **Fragment 3:** PCR with the primers cAcmA_fwd and cAcmA_rev with cAcMA as template. Purified fragments were joined via NEBuilder HiFi DNA Assembly

**pNZ8149_PLG**

**Fragment 1:** pNZ8149 was digested with NcoI HF and SpeI HF and the fragment of 2523 was gel-purified **Fragment 2:** PCR was done with primers Usp_45__fwd and Usp_45__flag_tag_rev with pNZ8149_USP_45__sfGFP plasmid as template. **Fragment 3:** PCR using primers Nanobody_fwd2 and Nanobody_RV_cAcmA 2 with H11D4 block as template. **Fragment 4: PCR using the primers** cAcmA_fwd2 and cAcMA_rev2 with cAcMA as template. Purified fragments were joined via NEBuilder HiFi DNA Assembly

**pNZ8149_PLA**

**Fragment 1:** pNZ8149_PLG digested with SphI/XbaI**,** the fragment of 2628 pb was gel purified. **Fragment 2:** PCR with H11-D4_fwd10 and H11-D4_rev10 using with H11D4 block as template. **Fragment 3:** PCR with the primers cAcma_fwd10 and cAcma_rev10 using cAcmA as template. Purified fragments were joined via NEBuilder HiFi DNA Assembly

**pNZ8149_PLB**

**Fragment 1:** pNZ8149_PLG digested with SpeI/SphI**,** the fragment of 3280pb was gel purified. **Fragment 2:** with H11-D4_fwd10 and H11-D4_rev10 using H11H4 block. Purified fragments were joined via NEBuilder HiFi DNA Assembly

**pNZ8149_PLC**

**Fragment 1:** pNZ8149 was digested with NcoI HF and SpeI HF and the fragment of 2523 was gel purified **Fragment 2:** PCR using primers Usp_45__fwd and Usp_45__flag_tag_rev with pNZ8149_USP_45__sfGFP plasmid as template. **Fragment 3:** PCR using primers Nanobody_fwd2 and Nanobody_RV_cAcmA 2 with pNZ8149_PLG as template. **Fragment 4:** PCR using the primers cAcmA_fwd2 and cAcMA_rev2 with cAcmA as template. Purified fragments were joined via NEBuilder HiFi DNA Assembly

**pNZ8149_PLD**

**Fragment 1:** pNZ8149_PLG digested with SpeI/SphI**,** the fragment of 3280pb was gel purified. **Fragment 2:** H11D4+linker. Purified fragments were joined via NEBuilder HiFi DNA Assembly

**pNZ8149_PLH**

**Fragment 1:** pNZ8149_PLG digested with SphI/XbaI**,** the fragment of 2628 pb was gel purified. **Fragment 2:** PCR with H11-D4_fwd10 and nanobody_rev13 using pNZ8149_PLB as template. **Fragment 3:** PCR with the primers cAcmA_linker_fwd14 and cAcmA_linker_rev14 using pNZ8149_PLD as template. Purified fragments were joined via NEBuilder HiFi DNA Assembly

**pNZ8149_PLJ**

**Fragment 1:** pNZ8149_PLG digested with SphI/XbaI**,** the fragment of 2628 pb was gel purified. **Fragment 2:** PCR with nanobody_fwd13 and nanobody_rev13 using pNZ8149_PLG as template. **Fragment 3:** PCR with the primers cAcmA_linker_fwd14 and cAcmA_linker_rev14 using pNZ8149_PLD as template. Purified fragments were joined via NEBuilder HiFi DNA Assembly

**pNZ8149_PLK**

**Fragment 1:** pNZ8149_PLG digested with SpeI/SphI**,** the fragment of 3280 pb was gel purified. **Fragment 2:**H11H4 block. Purified fragments were joined via NEBuilder HiFi DNA Assembly

**
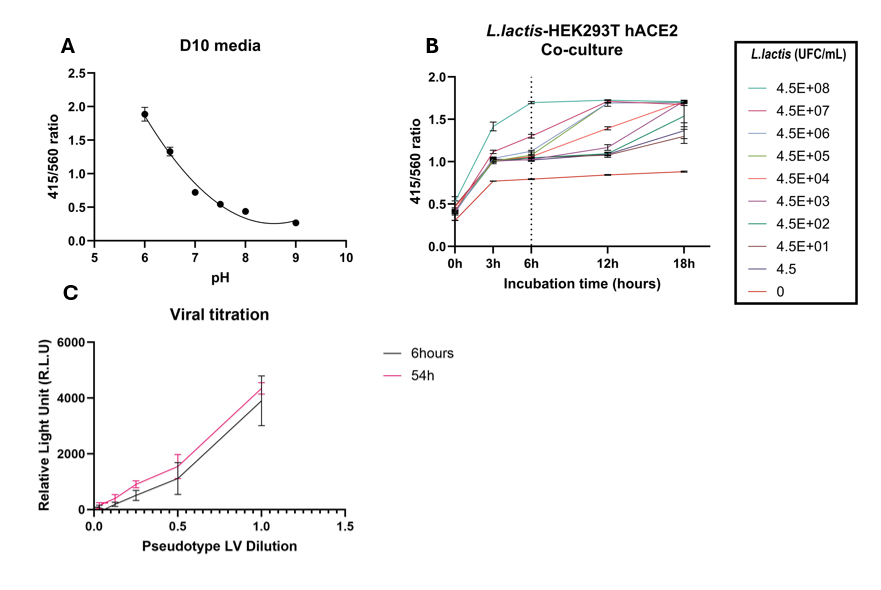
**

**Supplementary Fig. 2.** Adaptation in neutralization assay. **A.** Spectrometric determination of the pH in D10 media. The 415/560 ratio can predict pH changes. **B.** Effect of L. lactis concentration on pH in co-culture with HEK293T-hACE2 cells. At 6h incubation, the concentrations 4.5x10^5^ or lower have a pH near physiological conditions (6.92). This incubation was used in the following assays. **C.** Adaptation of Incubation time in spike-pseudotype lentivirus infection. HEK293T-hACE2 Cells were incubated for 54h with the spike-pseudotype lentivirus following the original protocol, in contrast with the adaptation where the cells were incubated for 6h with the spike-pseudotype lentivirus and 48h of incubation in D10 media with antibiotics. Viral infection was quantified by the expression of luciferase in human cells (RLU readout). Both protocols show similar trends in a viral titration assay. The spike-pseudotype lentivirus used in this assay was produced by BEI (**NR-53818).**

**
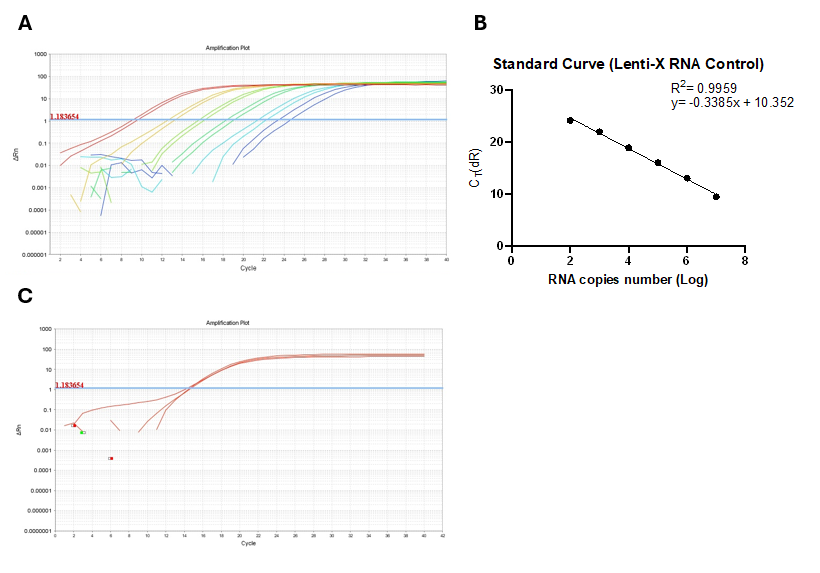
**

**Supplementary Fig. 3.** Quantification of Pseudo type lentivirus using RTqPCR. **A.** Lent-X RNA control template titration. qRT-PCR reactions of the positive control are visualized in amplification plots **B**. Lent-X RNA control standard curve. Positive control shows a strong correlation between the RNA copies number and C_T._  **C.** Amplification plot of purified pseudotype lentivirus RNA.

**SUPPLEMENTARY DATA REFERENCES**

(1) Overkamp, W.; Beilharz, K.; Detert Oude Weme, R.; Solopova, A.; Karsens, H.; Kovács, Á. T.; Kok, J.; Kuipers, O. P.; Veening, J.-W. Benchmarking Various Green Fluorescent Protein Variants in Bacillus Subtilis, Streptococcus Pneumoniae, and Lactococcus Lactis for Live Cell Imaging. *Appl Environ Microbiol* **2013**, *79* (20), 6481–6490. https://doi.org/10.1128/AEM.02033-13.

(2) Davarpanah, E.; Seyed, N.; Bahrami, F.; Rafati, S.; Safaralizadeh, R.; Taheri, T. Lactococcus Lactis Expressing Sand Fly PpSP15 Salivary Protein Confers Long-Term Protection against Leishmania Major in BALB/c Mice. *PLoS Negl Trop Dis* **2020**, *14* (1), e0007939. https://doi.org/10.1371/journal.pntd.0007939.
